# The Discovery of Data-Driven Temporal Dietary Patterns and a Validation of Their Description Using Energy and Time Cut-Offs

**DOI:** 10.3390/nu14173483

**Published:** 2022-08-24

**Authors:** Luotao Lin, Jiaqi Guo, Yitao Li, Saul B. Gelfand, Edward J. Delp, Anindya Bhadra, Elizabeth A. Richards, Erin Hennessy, Heather A. Eicher-Miller

**Affiliations:** 1 Department of Nutrition Science, Purdue University, West Lafayette, IN 47906, USA; 2 School of Electrical and Computer Engineering, Purdue University, West Lafayette, IN 47906, USA; 3 Department of Statistics, Purdue University, West Lafayette, IN 47906, USA; 4 School of Nursing, Purdue University, West Lafayette, IN 47906, USA; 5 Friedman School of Nutrition Science and Policy, Tufts University, Boston, MA 02111, USA

**Keywords:** temporal pattern, dietary pattern, energy intake, obesity, machine learning

## Abstract

Data-driven temporal dietary patterning (TDP) methods were previously developed. The objectives were to create data-driven temporal dietary patterns and assess concurrent validity of energy and time cut-offs describing the data-driven TDPs by determining their relationships to BMI and waist circumference (WC). The first day 24-h dietary recall timing and amounts of energy for 17,915 U.S. adults of the National Health and Nutrition Examination Survey 2007–2016 were used to create clusters representing four TDPs using dynamic time warping and the kernel k-means clustering algorithm. Energy and time cut-offs were extracted from visualization of the data-derived TDPs and then applied to the data to find cut-off-derived TDPs. The strength of TDP relationships with BMI and WC were assessed using adjusted multivariate regression and compared. Both methods showed a cluster, representing a TDP with proportionally equivalent average energy consumed during three eating events/day, associated with significantly lower BMI and WC compared to the other three clusters that had one energy intake peak/day at 13:00, 18:00, and 19:00 (all *p* < 0.0001). Participant clusters of the methods were highly overlapped (>83%) and showed similar relationships with obesity. Data-driven TDP was validated using descriptive cut-offs and hold promise for obesity interventions and translation to dietary guidance.

## 1. Introduction

The Dietary Guidelines for Americans, 2020–2025 [1] emphasizes the importance of a healthy dietary pattern rather than focusing on nutrients or foods in isolation. Dietary patterns are defined as “the quantities, proportions, variety, or combination of different foods, drinks and nutrients in diets, and the frequency with which they are habitually consumed” [2]. Therefore, dietary patterns include not only foods and their components such as energy, but also the behaviors inherent to dietary intake such as when eating and drinking occur. Yet, little attention has been given to the specific timing of dietary intake, perhaps due to the methodological difficulty of patterning temporal data. Only a few studies [3,4,5,6] have investigated the frequency and timing of eating and even fewer studies [7,8] have incorporated multiple aspects of dietary patterns, such as energy, frequency, and the specific timing of dietary intake, despite evidence of a link to dietary quality and ultimately, health. Data-driven methods were recently applied to create temporal dietary patterns (TDPs) incorporating timing and amount of energy intake over 24-h [3,4,5,6]. These studies showed that TDPs were significantly associated with obesity-related health indicators including body mass index (BMI) and waist circumference (WC) [3,4]. For example, participants with energy-equivalent and evenly distributed eating occasions throughout the day had higher diet quality [5], lower mean BMI and odds of obesity, and smaller WC [3] than those with other temporal dietary patterns exhibiting one energy intake peak sometime in the day. However, since data-driven methods that are based on the true nature of the behaviors were used, the patterns that emerged had no guiding description or indication of adherence to recommendations to explain the resulting patterns or the constraints of inclusion. The patterns emerging from such clusters may be difficult to describe and capture latent characteristics. Therefore, an interpretation describing these data-derived TDPs may not have similar relationships with obesity and should be validated to ensure accuracy. 

The purpose of this study was (1) to create data-driven TDPs and determine their relationships to BMI and WC; (2) then to extract the pattern characteristics using energy and time cut-offs based on visualizing the patterns and assess the concurrent validity of the cut-off-derived TDPs by determining the percentage of overlap in cluster membership and determining the cut-off TDPs relationships with BMI and WC. The hypothesis is that the strength of the relationship of TDPs based on energy intake and time cut-offs with BMI and WC is similar to the relationship of TDPs created using data-driven methods with BMI and WC, and participant membership to the various pattern clusters is highly overlapping between the similar cut-off and data-driven TDP clusters.

## 2. Materials and Methods

### 2.1. Participants and Data Set

Participants of the study were drawn from the National Health and Nutrition Examination Survey (NHANES) 2007–2016. NHANES is a National Center for Health Statistics (NCHS) conducted survey containing interviews and a physical health examination to quantify the health and nutritional status of U.S. adults and children. Voluntary participation is invited after selection based on location, characteristics, and randomness. Participants’ sociodemographic characteristics, including age, sex, race, ethnicity, and poverty-to-income ratio (PIR), were collected during the in-person household interview. Anthropometric measurement, including height, weight, and WC, and the first dietary recall interview were collected during the physical health examination. The NCHS Research Ethics Review Board approved this survey and all the participants consented to completing the survey [9].

**Figure 1:**
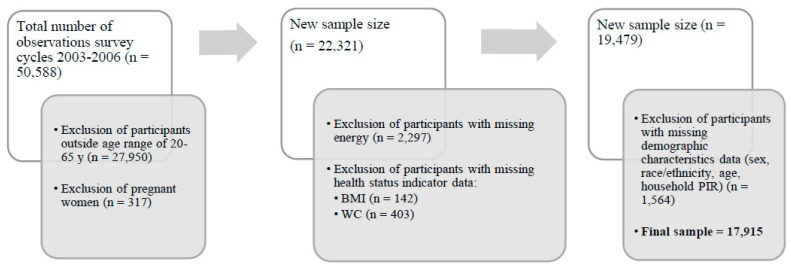
Flow chart representing sample size attrition and reason for exclusion. Final sample is this study sample that meet all the inclusion criteria. WC: waist circumference. BMI: body mass index.

NHANES 2007–2016 were used because data were the most recently available when the study was initiated. The sample included non-pregnant U.S. adults aged 20–65 years with reliable first-day 24-h dietary recall data and complete anthropometric measurements. The temporal dietary behaviors of pregnant women and participants outside of the age range are expected to exhibit unique life stage patterns and were excluded. Participants with missing sociodemographic and anthropometric data were also excluded. Therefore, the analytic sample included 17,915 participants (Figure 1).

### 2.2. Dietary Data Assessment

The USDA Automated Multiple-Pass Method was included in NHANES to collect the 24-h dietary recall data [10], including the time, amount, and type of foods and beverages consumed, and detailed food descriptions [11]. Valid 24-h dietary recalls that met the NHANES criteria [11] with non-zero energy intake were used in this study. Each participant’s energy intake for all reported foods and beverages was determined using the USDA Food and Nutrient Database for Dietary Studies (FNDDS) for 2007–2008 data (USDA FNDDS, version 4.1), 2009–2010 data (USDA FNDDS, version 5.0), 2011–2012 data (USDA FNDDS 2011–2012), 2013–2014 data (USDA FNDDS 2013–2014), and 2015–2016 data (USDA FNDDS 2015–2016) [12]. The time duration of the eating occasions was not available in NHANES, thus 15 min/occasion was applied at each time of reported intake based on a previous study [13] where reported energy at a time was divided by 15 min to determine the energy per minute for each minute within the 15-min eating occasion.

### 2.3. Anthropometric Measurement

Standing height and WC were measured in centimeters using a stadiometer and measuring tape, respectively. Weight was measured in kilograms using a digital weight scale [14,15,16]. BMI was calculated as a person’s weight in kilograms divided by the square of their height in meters [17]. 

### 2.4. Measures for Covariates

Survey year, sex, age group, race, ethnicity, PIR, and energy misreporting were used as covariates to adjust the regression models that evaluated the relationship of the TDPs to BMI and WC. Survey year included years 2007–2008, 2009–2010, 2011–2012, 2013–2014, and 2015–2016. Sex was classified as male and female. Race and ethnicity were classified as Mexican American, other Hispanic, non-Hispanic white, non-Hispanic black, and other including multi-race. PIR is the ratio of family income-to-poverty and was classified as 0–0.99 (under poverty threshold), 1–1.99, 2–2.99, 3–3.99, 4–4.99, and ≥5 [18]. Energy misreporting was considered a potential confounder to the relationships evaluated and was determined by calculating total energy intake divided by estimated energy requirement (EER) [19,20,21], where EER was derived based on dietary reference intake equations for adults according to the Institute of Medicine [22]. The NCHS assigns weights to participants in the NHANES based on their selection. Weights were constructed when combining survey cycles 2007–2016 and used in the models, thus the results are representative of the US civilian, noninstitutionalized population at the midpoint of the 10 years of data included in the study [23]. The survey design of NHANES included stratification and clustering, which were both accounted for in the regression models according to NCHS guidelines to improve the precision of survey estimates [24].

### 2.5. Creating TDPs through Data-Driven Method

A detailed description of the methods for creating the data-derived TDPs has been published previously [3,6]. Briefly, participants’ first 24-h dietary recall was considered as a time series of 24 h × 60 min = 1440 min with each entry representing the absolute amount of energy intake during that minute. Distance-based clustering analysis with a dynamic time warping (DTW)-type distance measure was used to create the TDPs. DTW optimally matches the eating events for each pair of participants in the sample by minimizing a weighted sum of the squared differences between the time and energy intakes of the respective participant’s eating events. A weight parameter is used to control the matching by penalizing the time differences relative to the energy uptake differences to avoid pathological matchings (such as matching morning to late night dietary intake), and this variation of DTW is denoted as modified DTW (MDTW) [25]. Then, the distance measure of diet was coupled with the kernel k-means algorithm [26] to partition the ensemble of time series into different clusters to develop TDPs without predetermined standards or cut-offs. The purpose of using the kernel k-means algorithm is to generate TDPs where the dietary intakes are similar within a cluster and more dissimilar between clusters. The number of clusters which partition the participants into mutually exclusive clusters was based first on internal criteria related to the variance and consistency of clusters including silhouette index and Dunn index [27,28] where k = 3 and k = 4 yielded the best results (Table 1). Using these criteria, a high value indicates that the participant’s temporal dietary behavior is well matched to its own cluster and poorly matched to neighboring clusters. 

Next, the number of clusters was evaluated by external criteria associated with the visualization, time and energy differences among the clusters, and health outcome analysis as described in Section 2.9 where k = 4 was optimal. External criteria were also used to optimize the weight parameter in MDTW.

### 2.6. Visualization of TDPs through Data-Driven Method

**Figure 2:**
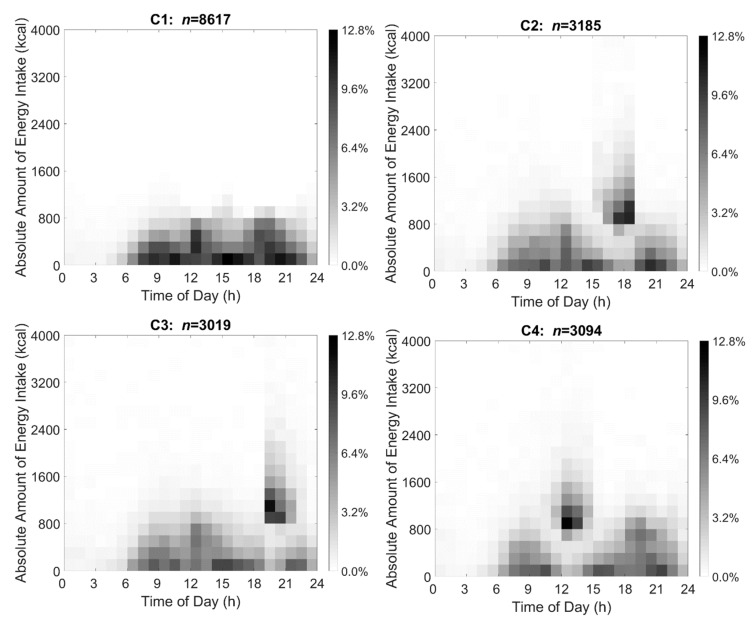
Energy intake heat maps representing four distinct TDPs generated based on data-driven methods for U.S. adults 20–65 years as drawn from NHANES 2007–2016 (*n* = 17,915). Absolute energy intake ranges from 0 to 4000 kcal (*y*-axis) while timing of intake ranges from 00:00 to 24:00 at hourly increments (*x*-axis) for non-pregnant U.S. adults 20–65 years. The proportion of participants in each cluster reporting energy intake is shown through shading ranging from 0.0% to 12.8% of participants in the 4 TDP clusters. Darker shading represents a greater percentage of participants in the cluster reporting the same amount of energy intake at that time. C1, cluster 1; C2, cluster 2; C3, cluster 3; C4, cluster 4; TDP, temporal dietary pattern.

Based on the criteria above, β = 40 generated the best TDPs. The visualization of the distribution of dietary intake in each of four clusters is illustrated using heat maps in Figure 2. The *x* axis indicates time ranging from 00:00 to 24:00, and the *y*-axis shows absolute energy intake ranging from 0 to 4000 kcal. The proportion of individuals in each cluster reporting dietary intake at a certain time and amount of energy is represented through shading and ranges from 0.0% to 12.8% in the 4 TDP clusters. Darker shading represents that a greater percentage of participants in the cluster reported the same amount of energy intake at that time.

### 2.7. Creating TDPs through Cut-Off Method

The heat map visualizations of the data-derived TDPs were used to describe the data and create the energy and time cut-offs. Specifically, shading indicating the proportion of the clusters with energy intake at the various hourly times were observed to find cut-points where the majority of energy intake and eating events occurred for each cluster. Furthermore, cut-offs were drawn both to describe each cluster independently and together in order that mutually exclusive clusters could be created. Based on Figure 2, no more than 800 kcal at any one eating event was used as the energy cut-off to distinguish cluster 1 from the other 3 clusters, meaning that participants whose energy intake was less than 800 kcal at any eating event during the day would be included in cluster 1. Next, for the remaining participants, the visualization in Figure 2 was used to determine if the participant’s highest energy intake occurred between 5:00 and 15:00 when the participant was assigned to cluster 4; if the participant’s highest energy intake occurred between 15:00 and 19:00 when the participant was assigned to cluster 2; if the participants’ highest energy intake occurred after 19:00 when the participant was assigned to cluster 3; or in the case that the participant had more than 1 highest energy intake during the day, the participant was assigned to cluster 1.

### 2.8. Visualization of TDPs through Cut-Off Method

**Figure 3:**
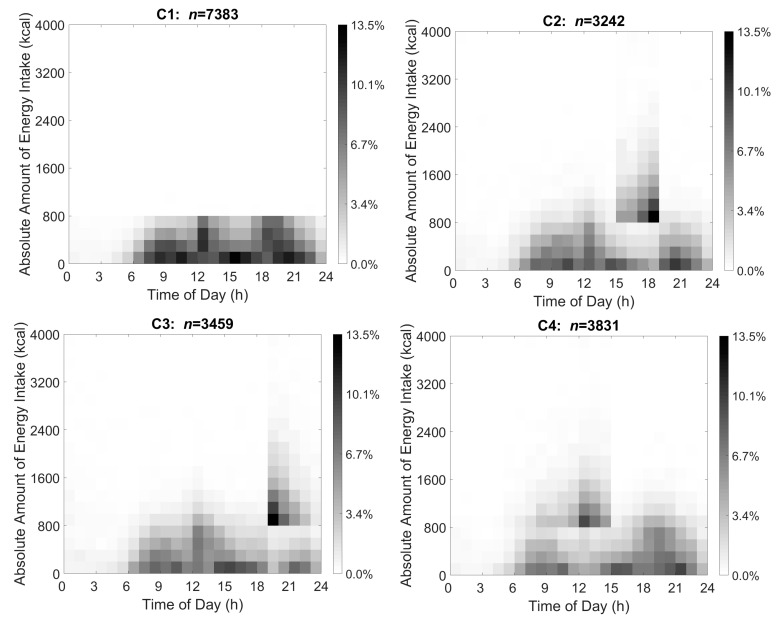
Energy intake heat maps representing four distinct TDPs generated based on the cut-off method for U.S. adults 20–65 years as drawn from NHANES 2007–2016 (*n* = 17,915). Absolute energy intake ranges from 0 to 4000 kcal (*y*-axis) while timing of intake ranges from 00:00 to 24:00 at hourly increments (*x*-axis) for non-pregnant U.S. adults 20–65 years. The proportion of participants in each cluster reporting energy intake is shown through shading ranging from 0.0% to 13.5% of participants in the 4 TDP clusters. Darker shading represents a greater percentage of participants in the cluster reporting the same amount of energy intake at that time. C1, cluster 1; C2, cluster 2; C3, cluster 3; C4, cluster 4; TDP, temporal dietary pattern.

Based on the data-driven TDP visualizations, the chosen cut-offs were used to generate cut-off-derived TDPs. The new cut-off-derived clusters were also visualized using heat maps and these TDS are shown in Figure 3. Similar to Figure 2, the *x*-axis indicates time ranging from 00:00 to 24:00, and the *y*-axis shows absolute energy intake ranging from 0 to 4000 kcal. The proportion of individuals in each cluster reporting dietary intake at a certain time and amount of energy is represented through shading and ranges from 0.0% to 13.5% in the 4 TDP clusters. The darker shading represents that a greater percentage of participants in the cluster reported the same amount of energy intake at that time.

### 2.9. Statistical Analysis

The Rao-Scott modified chi-square test was used to determine significant differences among clusters by characteristics including survey year, age group, sex, race/ethnicity, PIR, and BMI. Percent of participant overlap between the data-driven and cut-off-derived clusters representing a similar pattern was calculated. TDPs’ relationships with health indicators (BMI and WC) were assessed using adjusted multivariate linear regression. Residual plots and outliers were checked. Models using BMI and WC as health status indicators were adjusted for survey year, age group, sex, race/ethnicity, PIR, and energy misreporting. The Tukey–Kramer adjustment was made for multiple comparisons. Adjusted *p* < 0.05 for comparisons among clusters was considered statistically significant. SAS version 9.4 (SAS Institute Inc., Cary, NC, USA) and R version 4.1.1 (RStudio, Inc., Boston, MA, USA) were used to complete the analysis.

## 3. Results

### 3.1. Characteristics of Participants in the TDPs Clusters

The characteristics of the participants in the four clusters of TDP generated through two methods are shown in the Table 2.

### 3.2. Overlap between the Data-Driven Method and Cut-Off Method

The data-driven and cut-off TDPs generated four clusters with similar patterns. About 83.3%, 87.1%, 92.0%, and 89.1% of the participants in the cut-off-derived TDP clusters overlapped with participant membership in the data-driven TDP clusters. Compared with the other three clusters, the energy intake in cluster 1 was moderate, specifically, each of the main energy intake events included energy at less than 800 kcal. However, clusters 2, 3, and 4 all had an energy intake peak (reaching 4000 kcal) at different times during the day. Cluster 2’s energy intake peak was 15:00–19:00, cluster 3’s energy intake peak was after 19:00, and cluster 4’s energy intake peak was before 15:00.

### 3.3. Associations of TDPs with BMI and WC

The TDPs that were generated by both methods were significantly associated with BMI and WC. Participants in cluster 1 derived from both methods had significantly lower mean BMI and smaller mean WC compared to participants of clusters 2, 3, and 4 (Table 3 and Table 4). The greatest significant difference in mean BMI and mean WC were present between clusters 1 and 4 (β = −3.3 ± 0.2, R^2^ = 0.12) and clusters 1 and 3 (β = −8.2 ± 0.5 cm, R^2^ = 0.17) using the data-driven method and clusters 1 and 3 (β = −3.1 ± 0.2, R^2^ = 0.12 and β = −7.9 ± 0.4 cm, R^2^ = 0.17) using the cut-off method.

## 4. Discussion

The results of this study showed TDPs linked to BMI and WC though a data-driven method which were then used to extract and validate a time and energy-based interpretation of the patterns from the visualization of the data-driven TDPs by showing their similar significant relationship with obesity and percentage of overlap among cluster membership using both methods. To the best of our knowledge, this is the first study that created temporal lifestyle patterns using a machine learning method and then extracted a practical interpretation of the patterns that was also validated against U.S. weight outcomes, which will add to the evidence of the link between multidimensional dietary patterns and health. The mean differences in BMI and WC associated with TDPs were not only statistically significant but also clinically meaningful [29,30], which indicates that the timing and amount of dietary intake can be a potential important health exposure to predict and prevent obesity. Since the practical interpretation extracted from the visualization of the data-derived TDPs was similarly linked to the obesity-related indicators and the overlapping cluster membership rate was also shown, the description of the patterns is a validated interpretation of the TDPs. The evidence provides a basis that data-driven methods may be used to find and extract practically translatable TDPs, a topic that is relevant to the timing of dietary intake and highlighted as a question under consideration for the 2025 Dietary Guidelines for Americans’ scientific committee to address [31].

Findings from this study show that three evenly spaced, energy balanced eating occasions throughout the day are significantly associated with lower BMI and smaller WC compared to the TDPs that have one energy intake peak at different times throughout the day, which is supported by previous studies [3,4]. In addition, this study also showed that participants in cluster 4 that have an energy intake peak around 12:00 have significant higher BMI and larger WC compared to those in cluster 1. This finding is similar to a previous study where overweight or obese adults reported approximately four eating occasions a day, with the peak number of eating occasions occurring around 12:30 [32]. The pattern of cluster 2 with an energy intake peak after 15:00 and significantly higher BMI and larger WC compared to cluster 1, may have similarities with other findings. A previous study [33] showed that late lunch eaters lost significantly less weight and had slower rates of losing weight compared to early eaters after a 20-week intervention even though both groups had similar habitual energy intakes, and total energy expenditure.

Furthermore, the pattern of participants in cluster 3 with the highest energy intake peak at night (after 19:00), had significantly higher BMI and larger WC than those in cluster 1. This finding is aligned with a previous U.S.-based study [34], Japanese-based studies [35,36], Malaysian-based study [37] and Swedish-based study [38], all showing that late-night eating is associated with higher risk of obesity. High energy intake in the evening may be related to night eating syndrome [39], included in the fifth edition of the Diagnostic and Statistical Manual of Mental Disorders and identified as an eating disorder related to dysfunction of the circadian system. One of the reasons that night eating syndrome is significantly associated with obesity may be due to decreased diet-induced thermogenesis after dinner, which may lead to less energy expenditure and potential weight gain. This may also be because that the circadian system increases the glucagon production and reduces insulin production in anticipation of midnight fasting. Melatonin, a hormone signaling night in the circadian system, may further decrease the release of insulin at night. Thus late-night eating may cause a higher blood glucose rise and pose a risk for type 2 diabetes [40,41]. Late night eating is also significantly associated with increased energy intake [42] and may be a risk factor for obesity [43]. 

One of the biggest challenges in this study was to interpret the visualization of different clusters generated by the data-driven method. Unlike traditional clustering methods, DTW was used to develop TDPs in this study. DTP uses an elastic distance measure that can find the optimal matching paths among eating events of every pair of participants and quantify the pairwise distances between participants where the matched path is minimized. In addition, the kernel k-means algorithm was used to objectively divide participants into different TDPs based on the distances calculated from DTW. Since this objective method did not include predetermined standards or criteria for the temporal dietary patterns, the characteristics of the patterns of each of the TDPs that are generated by the data-driven method are not apparent. Visualization can be used to observe what the time, energy, and proportion of the group’s distributions look like and extract the temporal dietary behavior characteristics from each TDP. Previous studies also used visualizations to capture each TDPs’ characteristics [3,4,44] in either a DTW method coupled with the kernel k-means algorithm or a latent class analysis approach. Both methods were also previously used to identify unique and unknown patterns according to different observed indicators from multiple layers of data. Other studies used principal components to describe the dietary pattern [45,46]. In these principal component analyses, the major contributing food items or groups were used to describe the sentinel characteristics of the dietary patterns. However, the interpretation of the dietary patterns from these various methods only extracted certain factors including food, time, or amount of energy. Yet, other factors or characteristics of the patterns may be important. It is difficult to know whether other unobserved or observed factors should be prioritized as sentinel characteristics to describe the commonalities of the patterns, representing further needs to be addressed in future studies. Yet, validation of the pattern interpretation is critical to determine whether the selected factors to describe the patterns do indeed yield a similar cluster of participants and relationship with health. 

The need for validation of data-derived patterns is in contrast with more traditional index-derived patterns or model-based patterns that need no such validation as their interpretation is already apparent and dependent on preconceived criteria. For example, the Healthy Eating Index was created based on scoring linked to proportions of food groups as recommended in the Dietary Guidelines for Americans. Data-driven clusters have no similar criteria from which diets are judged or ranked and leave the interpretation of the patterns created through data-driven methods open to investigators to subjectively describe. The results of this study show that this limitation can be overcome by extracting descriptions based on visualizations and then validating these interpretations. Based on the authors knowledge, this is the first study that evaluates and validates a data-driven patterning interpretation through membership overlap and associations with obesity-related health indicators. The results showed that cut-off-derived clusters highly overlapped with data-driven clusters and demonstrated no differences in strength or pattern relationships with obesity-related indicators between the two methods. Therefore, although interpretation of the patterns has been a limitation for data-derived methods, it can be addressed and removed.

Considering the cross-sectional study design, the results cannot be used to infer causation. In addition, dietary data are from one weekday dietary recall, and data may not represent participants’ regular patterns. However, a single 24-h dietary recall may be considered to be representative to estimate the general dietary pattern if days of the week of dietary recalls are evenly selected [47]. Moreover, smaller and specific TDPs, such as night shift patterns or intermittent fasting, may exist but are not observed since these patterns may be combined with other patterns preventing observation of their unique temporal characteristics. 

The results provide evidence that data-driven methods have a high potential to discover patterns for which practical interpretations can be extracted and validated and easily translated to practical temporal dietary guidance to prevent obesity such as in the Dietary Guidelines for Americans. In addition, the development of time-based dietary intake translation may also be useful in detecting and prompting interventions to modify daily temporal patterns, potentially integrating other lifestyle behaviors including physical activity and sleep, and informing individualized, precision nutrition.

## 5. Conclusions

Four cut-off-derived clusters based on the visualization of data-driven clusters highly overlapped with data-driven clusters and showed no differences in strength or pattern relationships with obesity. The results provide evidence that data-driven methods have a high potential to discover patterns that are easily translatable to practical temporal dietary guidance to prevent obesity such as in the Dietary Guidelines for Americans. The developed time-based dietary intake translation may also be useful in detecting and prompting interventions to modify daily temporal patterns, potentially integrating other lifestyle behaviors including physical activity and sleep, and informing individualized, precision nutrition.

## Figures and Tables

**Table 1:** Silhouette and Dunn index values ^1^ of internal cluster variance and consistency for three to seven clusters partitioning U.S. adults 20–65 year as drawn from NHANES 2007–2016 (*n* = 17,915).

	Cluster K Partitions				
	K = 3	K = 4	K = 5	K = 6	K = 7
Silhouette Index	0.27	0.25	0.19	0.18	0.15
Dunn Index	0.07	0.05	0.02	0.01	0.04

^1^ higher values indicate better clustering.

**Table 2:** Characteristics of clusters representing data-driven TDPs and cut-off-derived TDPs of U.S. adults 20–65 year as drawn from the NHANES, 2007–2016.

		Data-Driven TDPs				Cut-Off-Derived TDPs			
Characteristics	Total (*n*)	Cluster 1 ^1^	Cluster 2 ^1^	Cluster 3 ^1^	Cluster 4 ^1^	Cluster 1 ^1^	Cluster 2 ^1^	Cluster 3 ^1^	Cluster 4 ^1^
Total	17,915	8617 (48.1)	3185 (17.8)	3019 (16.8)	3094 (17.3)	7383 (41.2)	3242 (18.1)	3459 (19.3)	3831 (21.4)
Survey year		*p*-value ^2^ = 0.43				*p*-value ^2^ = 0.17			
2007–2008	3591 (20.0)	1755 (20.4)	648 (20.3)	571 (18.9)	617 (19.9)	1518 (20.6)	670 (20.7)	651 (18.8)	752 (19.6)
2009–2010	3882 (21.7)	1898 (22.0)	692 (21.7)	644 (21.3)	648 (20.9)	1631 (22.1)	693 (21.4)	743 (21.5)	815 (21.3)
2011–2012	3441 (19.2)	1594 (18.5)	603 (18.9)	619 (20.5)	625 (20.2)	1361 (18.4)	604 (18.6)	711 (20.6)	765 (20.0)
2013–2014	3579 (20.0)	1757 (20.4)	628 (19.7)	606 (20.1)	588 (19.0)	1494 (20.2)	657 (20.3)	690 (19.9)	738 (19.3)
2015–2016	3422 (19.1)	1613 (18.7)	614 (19.3)	579 (19.2)	616 (19.9)	1379 (18.7)	618 (19.1)	664 (19.2)	761 (19.9)
Sex		*p*-value ^2^ < 0.0001 *				*p*-value ^2^ < 0.0001 *			
Male	8826 (49.3)	2884 (33.5)	1943 (61.0)	1987 (65.8)	2012 (65.0)	2346 (31.8)	1891 (58.3)	2190 (63.3)	2399 (62.6)
Female	9089 (50.7)	5733 (66.5)	1242 (39.0)	1032 (34.2)	1082 (35.0)	5037 (68.2)	1351 (41.7)	1269 (36.7)	1432 (37.4)
Race/Ethnicity		*p*-value ^2^ < 0.0001 *				*p*-value ^2^ < 0.0001 *			
Mexican American and Other Hispanic	4838 (27.0)	2341 (27.2)	896 (28.1)	739 (24.5)	862 (27.9)	1973 (26.7)	929 (28.6)	826 (23.9)	1110 (29.0)
Non-Hispanic white	7218 (40.3)	3310 (38.4)	1425 (44.7)	1262 (41.8)	1221 (39.5)	2901 (39.3)	1397 (43.1)	1432 (41.4)	1488 (38.8)
Non-Hispanic black and Other	5859 (32.7)	2966 (34.4)	864 (27.1)	1018 (33.7)	1011 (32.7)	2509 (34.0)	916 (28.3)	1201 (34.7)	1233 (32.1)
Age group (year)		*p*-value ^2^ < 0.0001 *				*p*-value ^2^ < 0.0001 *			
20–34	5761 (32.2)	2478 (28.8)	970 (30.5)	1147 (38.0)	1166 (37.7)	2071 (28.1)	1004 (31.0)	1348 (39.0)	1338 (34.9)
35–49	5920 (33.0)	2787 (32.3)	1120 (35.2)	978 (32.4)	1035 (33.5)	2364 (32.0)	1125 (34.7)	1107 (32.0)	1324 (34.6)
50–65	6234 (34.8)	3352 (38.9)	1095 (34.4)	894 (29.6)	893 (28.9)	2948 (39.9)	1113 (34.3)	1004 (29.0)	1169 (30.5)
Household PIR		*p*-value ^2^ = 0.013 *				*p*-value ^2 =^ 0.0005 *			
0–0.99	4154 (23.2)	2029 (23.5)	739 (23.2)	660 (21.9)	726 (23.5)	1729 (23.4)	763 (23.5)	744 (21.5)	918 (24.0)
1.00–2.99	4525 (25.3)	2234 (25.9)	802 (25.2)	716 (23.7)	773 (25.0)	1908 (25.8)	805 (24.8)	845 (24.4)	967 (25.2)
2.00–2.99	2567 (14.3)	1205 (14.0)	455 (14.3)	415 (13.7)	492 (15.9)	1027 (13.9)	468 (14.4)	454 (13.1)	618 (16.1)
3.00–3.99	1946 (10.9)	923 (10.7)	360 (11.3)	354 (11.7)	309 (10.0)	790 (10.7)	350 (10.8)	407 (11.8)	399 (10.4)
4.00–4.99	1425 (8.0)	649 (7.5)	270 (8.5)	253 (8.4)	253 (8.2)	566 (7.7)	278 (8.6)	292 (8.4)	289 (7.5)
≥5.00	3298 (18.4)	1577 (18.3)	559 (17.6)	621 (20.6)	541 (17.5)	1363 (18.5)	578 (17.8)	717 (20.7)	640 (16.7)

Abbreviations: NHANES, National Health and Nutrition Examination Survey; PIR, poverty to income ratio; TDPs, temporal dietary patterns. ^1^ Values are *n* (%). ^2^ Rao–Scott F adjusted χ^2^ *p*-value is a goodness-of-fit, one-sided test; statistical significance is indicated when *p* < 0.05. Analyses were adjusted for clustering and stratification. Sample weights were constructed and applied to the analysis as directed by the NCHS. Weights were rescaled in order that the sum of the weights matched the survey population at the midpoint of 2007–2016. Significance level: * adjusted *p* < 0.05.

**Table 3:** Adjusted regression model results for mean BMI (kg/m^2^) with clusters representing TDPs of U.S. adults 20–65 years as drawn from the NHANES, 2007–2016, generated based on data-driven methods and cut-off methods.

Adjusted Models ^1^	*n* (%)	BMI (kg/m^2^) ^2^	β ^3^ ± SE Compared to Cluster 2	95% CI	*p*-Value	β ^3^ ± SE Compared to Cluster 3	95% CI	*p*-Value	β ^3^ ± SE Compared to Cluster 4	95% CI	*p*-Value
**Data-Driven Methods**											
Cluster 1	8617 (48.1)	29.1 (0.1)	−3.0 ± 0.2	−3.7, −2.4	<0.0001 *	−3.3 ± 0.2	−3.8, −2.7	<0.0001 *	−3.3 ± 0.2	−3.9, −2.8	<0.0001 *
Cluster 2	3185 (17.8)	29.5 (0.1)				−0.2 ± 0.2	−0.8, 0.4	0.73	−0.3 ± 0.2	−0.9, 0.3	0.64
Cluster 3	3019 (16.8)	29.2 (0.1)							−0.0 ± 0.2	−0.5, 0.4	0.99
Cluster 4	3094 (17.3)	29.3 (0.1)									
**Cut-Off Methods**											
Cluster 1	7383 (41.2)	29.1 (0.1)	−2.9 ± 0.2	−3.5, −2.4	<0.0001 *	−3.1 ± 0.2	−3.6, −2.7	<0.0001 *	−2.9 ± 0.2	−3.4, −2.4	<0.0001 *
Cluster 2	3242 (18.1)	29.5 (0.1)				−0.2 ± 0.2	−0.7, 0.3	0.68	−0.0 ± 0.2	−0.6, 0.5	0.99
Cluster 3	3459 (19.3)	29.4 (0.1)							0.2 ± 0.2	−0.2, 0.6	0.59
Cluster 4	3831 (21.4)	29.1 (0.1)									

Abbreviations: BMI, body mass index; NHANES, National Health and Nutrition Examination Survey; SE, standard error; TDPs, temporal dietary patterns. ^1^ Models were adjusted for survey year, age group, sex, race/ethnicity, poverty to income ratio, and energy misreporting. ^2^ Values are mean (standard error of the mean). ^3^ ß represents the difference of mean BMI between two compared clusters. Least square means were used to calculate the differences in mean BMI. Significance level: * adjusted *p* < 0.05.

**Table 4:** Adjusted regression model results for mean WC (cm) with clusters representing TDPs of U.S. adults 20–65 years as drawn from the NHANES, 2007–2016, generated based on data-driven methods and cut-off methods.

Adjusted Models ^1^	*n* (%)	WC (cm) ^2^	β ^3^ ± SE Compared to Cluster 2	95% CI	*p*-Value	β ^3^ ± SE Compared to Cluster 3	95% CI	*p*-Value	β ^3^ ± SE Compared to Cluster 4	95% CI	*p*-Value
**Data-Driven Methods**											
Cluster 1	8617 (48.1)	97.7 (0.2)	−7.4 ± 0.6	−9.0, −5.9	<0.0001 *	−8.2 ± 0.5	−9.5, −6.9	<0.0001 *	−8.2 ± 0.5	−9.4, −6.9	<0.0001 *
Cluster 2	3185 (17.8)	100.1 (0.3)				−0.7 ± 0.6	−2.3, 0.7	0.55	−13.4 ± 1.7	−2.2, 0.8	0.62
Cluster 3	3019 (16.8)	99.4 (0.3)							−0.1 ± 0.4	−1.1, 1.2	0.99
Cluster 4	3094 (17.3)	99.3 (0.3)									
**Cut-Off Methods**											
Cluster 1	7383 (41.2)	97.5 (0.2)	−7.3 ± 0.5	−8.6, −5.9	<0.0001 *	−7.9 ± 0.4	−8.9, −6.9	<0.0001 *	−7.5 ± 0.4	−8.7, −6.4	<0.0001 *
Cluster 2	3242 (18.1)	100.0 (0.3)				−0.7 ± 0.5	−2.0, 0.7	0.56	−0.3 ± 0.5	−1.7, 1.2	0.97
Cluster 3	3459 (19.3)	99.6 (0.3)							0.4± 0.4	−0.6, 1.4	0.75
Cluster 4	3831 (21.4)	99.1 (0.3)									

Abbreviations: NHANES, National Health and Nutrition Examination Survey; TDPs, temporal dietary patterns; SE, standard error; WC, waist circumference. ^1^ Models were adjusted for survey year, age group, sex, race/ethnicity, poverty to income ratio, and energy misreporting. ^2^ Values are mean (standard error of the mean). ^3^ ß represents the difference of mean WC between two compared clusters. Least square means were used to calculate the differences in mean WC. Significance level: * adjusted *p* < 0.05.

## Data Availability

Data described in the manuscript are made publicly and freely available without restriction at https://www.cdc.gov/nchs/nhanes/index.htm (accessed on 2 June 2020). Analytic code is available upon request.

## References

[1] U.S. Department of Agriculture and U.S. Department of Health and Human Services Dietary Guidelines for Americans, 2020–2025. p. 164. https://www.dietaryguidelines.gov/sites/default/files/2020-12/Dietary_Guidelines_for_Americans_2020-2025.pdf.

[2] DietaryPatternsReport-FullFinal2.pdf. https://nesr.usda.gov/sites/default/files/2019-06/DietaryPatternsReport-FullFinal2.pdf.

[3] Aqeel M.M., Guo J., Lin L., Gelfand S.B., Delp E.J., Bhadra A., Richards E.A., Hennessy E., Eicher-Miller H.A. (2020). Temporal Dietary Patterns Are Associated with Obesity in US Adults. J. Nutr..

[4] Lin L., Guo J., Aqeel M.M., Gelfand S.B., Delp E.J., Bhadra A., Richards E.A., Hennessy E., Eicher-Miller H.A. (2022). Joint temporal dietary and physical activity patterns: Associations with health status indicators and chronic diseases. Am. J. Clin. Nutr..

[5] Eicher-Miller H.A., Khanna N., Boushey C.J., Gelfand S.B., Delp E.J. (2016). Temporal dietary patterns derived among the adult participants of NHANES 1999–2004 are associated with diet quality. J. Acad. Nutr. Diet..

[6] Khanna N., Eicher-Miller H.A., Boushey C.J., Gelfand S.B., Delp E.J. Temporal Dietary Patterns Using Kernel k-Means Clustering. Proceedings of the 2011 IEEE International Symposium on Multimedia.

[7] Huseinovic E., Winkvist A., Bertz F., Forslund H.B., Brekke H.K. (2014). Eating frequency, energy intake and body weight during a successful weight loss trial in overweight and obese postpartum women. Eur. J. Clin. Nutr..

[8] Hutchison A.T., Heilbronn L.K. (2016). Metabolic impacts of altering meal frequency and timing—Does when we eat matter?. Biochimie.

[9] NHANES-NCHS Research Ethics Review Board Approval. 9 November 2021. https://www.cdc.gov/nchs/nhanes/irba98.htm.

[10] AMPM-USDA Automated Multiple-Pass Method: USDA ARS. https://www.ars.usda.gov/northeast-area/beltsville-md-bhnrc/beltsville-human-nutrition-research-center/food-surveys-research-group/docs/ampm-usda-automated-multiple-pass-method/.

[11] NHANES Dietary Data. https://wwwn.cdc.gov/Nchs/Nhanes/Search/DataPage.aspx?Component=Dietary.

[12] Fndds Download Databases: USDA ARS. https://www.ars.usda.gov/northeast-area/beltsville-md-bhnrc/beltsville-human-nutrition-research-center/food-surveys-research-group/docs/fndds-download-databases/.

[13] Gibney M.J., Wolever T.M.S. (1997). Periodicity of eating and human health: Present perspective and future directions. Br. J. Nutr..

[14] Anthropometric Reference Data for Children and Adults; United States, 2007–2010. https://stacks.cdc.gov/view/cdc/12223.

[15] Anthropometric Reference Data for Children and Adults; United States, 2011–2014. https://stacks.cdc.gov/view/cdc/40572.

[16] Anthropometric Reference Data for Children and Adults: United States, 2015–2018. https://stacks.cdc.gov/view/cdc/100478.

[17] CDC Defining Adult Overweight and Obesity. Centers for Disease Control and Prevention, 3 May 2022. https://www.cdc.gov/obesity/basics/adult-defining.html.

[18] Bureau U.C. Poverty Thresholds. Census.gov. https://www.census.gov/data/tables/time-series/demo/income-poverty/historical-poverty-thresholds.html.

[19] Leech R.M., Worsley A., Timperio A., McNaughton S.A. (2018). The role of energy intake and energy misreporting in the associations between eating patterns and adiposity. Eur. J. Clin. Nutr..

[20] Wang J.B., Patterson R.E., Ang A., Emond J.A., Shetty N., Arab L. (2014). Timing of energy intake during the day is associated with the risk of obesity in adults. J. Hum. Nutr. Diet..

[21] Murakami K., Livingstone M.B.E. (2016). Associations between meal and snack frequency and overweight and abdominal obesity in US children and adolescents from National Health and Nutrition Examination Survey (NHANES) 2003–2012. Br. J. Nutr..

[22] Institute of Medicine (2002). Dietary Reference Intakes for Energy, Carbohydrate, Fiber, Fat, Fatty Acids, Cholesterol, Protein, and Amino Acids.

[23] NHANES Tutorials-Module 3-Weighting. https://wwwn.cdc.gov/nchs/nhanes/tutorials/module3.aspx.

[24] NHANES Tutorials-Module 2-Sample Design. https://wwwn.cdc.gov/nchs/nhanes/tutorials/module2.aspx.

[25] Eicher-Miller H.A., Gelfand S., Hwang Y., Delp E., Bhadra A., Guo J. (2020). Distance metrics optimized for clustering temporal dietary patterning among U.S. adults. Appetite.

[26] Dhillon I.S., Guan Y., Kulis B. Kernel k-means: Spectral clustering and normalized cuts. Proceedings of the Tenth ACM SIGKDD International Conference on Knowledge Discovery and Data Mining.

[27] Rousseeuw P.J. (1987). Silhouettes: A graphical aid to the interpretation and validation of cluster analysis. J. Comput. Appl. Math..

[28] Dunn J.C. (1974). Well-Separated Clusters and Optimal Fuzzy Partitions. J. Cybern..

[29] Bodegard J., Sundström J., Svennblad B., Östgren C.J., Nilsson P.M., Johansson G. (2013). Changes in body mass index following newly diagnosed type 2 diabetes and risk of cardiovascular mortality: A cohort study of 8486 primary-care patients. Diabetes Metab..

[30] Mulligan A.A., Lentjes M.A.H., Luben R.N., Wareham N.J., Khaw K.-T. (2019). Changes in waist circumference and risk of all-cause and CVD mortality: Results from the European Prospective Investigation into Cancer in Norfolk (EPIC-Norfolk) cohort study. BMC Cardiovasc. Disord..

[31] Dietary Guidelines Advisory Committee (2020). Scientific Report of the 2020 Dietary Guidelines Advisory Committee: Advisory Report to the Secretary of Agriculture and Secretary of Health and Human Services.

[32] Popp C.J., Curran M., Wang C., Prasad M., Fine K., Gee A., Nair N., Perdomo K., Chen S., Hu L. (2021). Temporal Eating Patterns and Eating Windows among Adults with Overweight or Obesity. Nutrients.

[33] Garaulet M., Gómez-Abellán P., Alburquerque-Béjar J.J., Lee Y.-C., Ordovás J.M., Scheer F.A.J.L. (2013). Timing of food intake predicts weight loss effectiveness. Int. J. Obes..

[34] Xiao Q., Garaulet M., Scheer F.A.J.L. (2019). Meal timing and obesity: Interactions with macronutrient intake and chronotype. Int. J. Obes..

[35] Kutsuma A., Nakajima K., Suwa K. (2014). Potential Association between Breakfast Skipping and Concomitant Late-Night-Dinner Eating with Metabolic Syndrome and Proteinuria in the Japanese Population. Scientifica.

[36] Yoshida J., Eguchi E., Nagaoka K., Ito T., Ogino K. (2018). Association of night eating habits with metabolic syndrome and its components: A longitudinal study. BMC Pub. Health.

[37] Mazri F.H., Manaf Z.A., Shahar S., Mat Ludin A.F., Karim N.A., Hazwari N.D.D., Kek Q.W., Basir S.M.A., Arifin A. (2021). Do Temporal Eating Patterns Differ in Healthy versus Unhealthy Overweight/Obese Individuals?. Nutrients.

[38] Berg C., Lappas G., Wolk A., Strandhagen E., Torén K., Rosengren A., Thelle D., Lissner L. (2009). Eating patterns and portion size associated with obesity in a Swedish population. Appetite.

[39] Cleator J., Abbott J., Judd P., Sutton C., Wilding H.J.P. (2012). Night eating syndrome: Implications for severe obesity. Nutr. Diabetes.

[40] Manoogian E.N.C., Wei-Shatzel J., Panda S. (2022). Assessing temporal eating pattern in free living humans through the myCircadianClock app. Int. J. Obes..

[41] Lopez-Minguez J., Gómez-Abellán P., Garaulet M. (2019). Timing of Breakfast, Lunch, and Dinner. Effects on Obesity and Metabolic Risk. Nutrients.

[42] Allison K.C., Ahima R.S., O’Reardon J.P., Dinges D.F., Sharma V., Cummings D.E., Heo M., Martino N.S., Stunkard A.J. (2005). Neuroendocrine Profiles Associated with Energy Intake, Sleep, and Stress in the Night Eating Syndrome. J. Clin. Endocrinol. Metab..

[43] Marshall H.M., Allison K.C., O’Reardon J.P., Birketvedt G., Stunkard A.J. (2004). Night eating syndrome among nonobese persons. Int. J. Eat. Disord..

[44] Leech R.M., Worsley A., Timperio A., McNaughton S.A. (2017). Temporal eating patterns: A latent class analysis approach. Int. J. Behav. Nutr. Phys. Act..

[45] Rasmussen M.A., Maslova E., Halldorsson T.I., Olsen S.F. (2014). Characterization of Dietary Patterns in the Danish National Birth Cohort in Relation to Preterm Birth. PLoS ONE.

[46] Chiu C.-J., Chang M.-L., Li T., Gensler G., Taylor A. (2017). Visualization of Dietary Patterns and Their Associations With Age-Related Macular Degeneration. Investig. Ophthalmol. Vis. Sci..

[47] Update on NHANES Dietary Data: Focus on Collection, Release, Analytical Considerations, and Uses to Inform Public Policy|Advances in Nutrition|Oxford Academic. https://academic.oup.com/advances/article/7/1/121/4524042.

